# Advances and challenges in immunotherapy for intrahepatic cholangiocarcinoma based on the tumour immune microenvironment

**DOI:** 10.1002/ctm2.70830

**Published:** 2026-09-11

**Authors:** Mengyao Chen, Shuofan Jin, Yuxuan Zhou, Jinkai Tong, Yufan Zhao, Quanjun Yang

**Affiliations:** ^1^ Department of Pharmacy Shanghai Sixth People's Hospital Affiliated Shanghai Jiao Tong University School of Medicine Shanghai China; ^2^ College of Food Science and Technology Shanghai Ocean University Shanghai China; ^3^ School of Biomedical Engineering Donghua University Shanghai China; ^4^ College of Clinical Pharmacy Shanghai Jiao Tong University School of Medicine Shanghai China

**Keywords:** biomarkers, FGFR2 fusion, IDH1 mutation, immune checkpoint inhibitors, intrahepatic cholangiocarcinoma, precision immunotherapy, tumour immune escape, tumour immune microenvironment

## Abstract

**Background:**

Intrahepatic cholangiocarcinoma (ICC) is a highly heterogeneous and aggressive malignancy originating from the intrahepatic bile duct epithelial cells. Despite advances in surgery, chemotherapy, targeted therapy, and immune checkpoint inhibitor (ICI)‐based regimens, durable disease control remains limited by primary or acquired resistance and marked biological heterogeneity.

**Main body:**

Immunotherapy, primarily based on ICIs, has shown clinical potential for advanced ICC; however, its activity is constrained by a highly immunosuppressive tumour microenvironment (TME), including desmoplastic stromal barriers, myeloid‐cell‐mediated suppression, impaired antigen presentation, cytokine‐ and chemokine‐driven immune exclusion, and metabolic remodeling. This review summarizes the tumour immune microenvironment (TIME) of ICC, with emphasis on cellular crosstalk among stromal, myeloid, and lymphoid compartments, extracellular matrix remodeling, metabolic constraints, and immune‐escape processes. We critically appraised first‐line chemoimmunotherapy evidence from advanced biliary tract cancer (BTC) trials and interpreted its relevance to ICC according to clinical evidence, tumour location, molecular subtype, and biomarker context. Biomarkers are discussed according to their current level of clinical validation, and molecular alterations, such as FGFR2 fusions, IDH1 mutations, and BAP1/ARID1A alterations, are considered in relation to immune phenotypes and mechanism‐guided combination hypotheses. Finally, we evaluated emerging approaches, including next‐generation checkpoint inhibitors, antibody–drug conjugates, bispecific antibodies, CAR‐T/CAR‐macrophage (CAR‐M)/CAR‐natural killer (CAR‐NK) cell therapies, cancer vaccines, and dendritic cell‐based interventions, with particular attention paid to antigen heterogeneity, stromal trafficking barriers, manufacturing complexity, and the limited maturity of ICC‐specific clinical evidence.

**Conclusions:**

By integrating the mechanism, clinical evidence, molecular subtype and translational limitations, this review provides a balanced framework for biomarker‐guided precision immunotherapy in ICC.

**Key Points:**

- The clinical evidence and limitations of immunotherapy for intrahepatic cholangiocarcinoma are critically evaluated, with emphasis on ICC‐specific interpretation of broader biliary tract cancer trials.
- The ICC tumour immune microenvironment is integrated into functional immune axes involving stromal, myeloid, lymphoid and metabolic regulation.
- Molecular subtypes and immune‐related biomarkers provide a framework for biomarker‐guided precision immunotherapy in ICC.
- Current ICC immunotherapy trials remain limited by heterogeneous designs, small ICC‐specific cohorts and immature clinical evidence.
- Emerging immunotherapies, including next‐generation checkpoint inhibitors, antibody‐drug conjugates, cellular therapies and vaccines, require ICC‐specific evaluation of biological barriers and clinical feasibility.

## Graphical abstract

This review integrates ICC‐specific and broader BTC evidence to define how desmoplastic stroma, immune‐cell crosstalk, metabolic remodelling, and molecular heterogeneity shape immunotherapy response and resistance. It highlights biomarker‐guided, mechanism‐based combinations while emphasizing the limited maturity of ICC‐specific evidence for emerging immunotherapies.

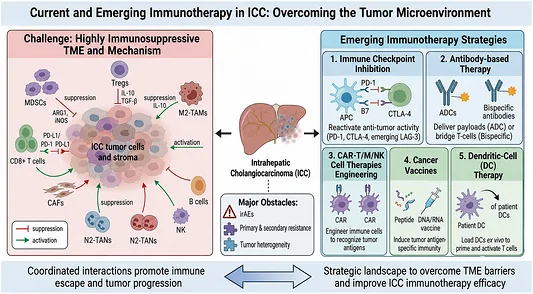

## 1 INTRODUCTION

Cholangiocarcinoma is a highly fatal malignant tumour of the digestive system that originates in bile duct epithelial cells. According to anatomical location, cholangiocarcinoma can be divided into intrahepatic cholangiocarcinoma (ICC), perihilar cholangiocarcinoma (pCC) and distal cholangiocarcinoma (dCC). ICC originates in the biliary tree proximal to the secondary intrahepatic bile ducts, whereas pCC and dCC arise from the extrahepatic biliary tract.^1^ These three subtypes differ not only in anatomical location but also in molecular features, immune contexture, and therapeutic sensitivity. ICC accounts for 8.2%–15.0% of primary liver malignancies, second only to hepatocellular carcinoma (HCC),^2^ and its incidence and mortality rates are increasing annually. The aetiology of ICC is complex, and most patients have no obvious early symptoms, making timely diagnosis difficult.^3^ Surgical resection remains the best curative treatment, but most patients miss the optimal surgical window at diagnosis and recurrence after resection remains frequent.^4^ Despite recent advances in systemic therapies, ICC remains a challenging malignancy with aggressive biological behaviour and limited long‐term disease control.^5^ Durvalumab‐ and pembrolizumab‐based regimens have improved survival in the broader BTC population, whereas IDH1 and FGFR2 inhibitors have provided opportunities for molecularly selected treatment strategies in subsets of cholangiocarcinoma patients.^6^, ^7^ Poor prognosis is closely associated with an immunosuppressive microenvironment.

The tumour microenvironment (TME) of ICC is composed of cellular and noncellular components. Cancer‐associated fibroblasts (CAFs), tumour‐associated macrophages (TAMs), tumour‐associated neutrophils (TANs), myeloid‐derived suppressor cells (MDSCs), natural killer (NK) cells, tumour‐infiltrating lymphocytes (TILs), dendritic cells (DCs), extracellular matrix (ECM), chemokines, extracellular vesicles (EVs), platelet‐derived growth factor (PDGF), and Notch signalling pathways interact with each other in multiple ways. These multiple components interact dynamically to form tumour‐promoting, angiogenesis‐promoting and immunosuppressive characteristics.^8^, ^9^, ^10^, ^11^, ^12^, ^13^, ^14^, ^15^, ^16^, ^17^, ^18^, ^19^ This complex multipathway process leads to the limited efficacy of current single therapies in most ICC patients and is an important factor that restricts clinical prognosis. A thorough understanding of the composition, regulatory network, and functions of the ICC immune microenvironment remains of urgent scientific significance and clinical value for breaking the current treatment bottleneck and developing new therapeutic strategies.

Recent reviews have provided valuable summaries of the CCA/ICC tumour immune microenvironment (TIME), immune cell infiltration, targeted therapy, and immunotherapy for BTC.^8^, ^14^, ^20^, ^21^ Building on these studies, this review emphasizes the current status of immunotherapy, TIME crosstalk, molecular subtype‐guided immunotherapy, emerging therapies, and related clinical trials. A persistent gap is that immune cell, stromal, and molecular findings are often presented in parallel, whereas clinical resistance is likely to emerge from their interactions. Therefore, this review adopts a mechanistic framework linking desmoplastic stroma, myeloid recruitment, lymphocyte dysfunction, metabolic stress, and molecular subtypes to treatment response. In particular, it places greater emphasis on the clinical interpretation of immunotherapy evidence in ICC, the stromal and myeloid barriers that shape the ICC TIME, and the relationship between molecular alterations, immune phenotypes, and treatment selection. First‐line immunochemotherapy evidence from advanced BTC trials has been interpreted in the context of ICC biology, considering tumour location, molecular subtype, and biomarker characteristics. Emerging immunotherapeutic strategies have been reviewed in the context of their current clinical maturity and potential relevance to ICC. Therefore, this review aims to systematically summarize current research on ICC immunotherapy, analyse the characteristics and regulatory mechanisms of the ICC immune microenvironment, and provide an updated overview of clinical evidence, molecular biomarkers, and emerging therapeutic strategies for future precision immunotherapy.

For this narrative review, relevant literature was identified through PubMed, Web of Science, EMBASE, Cochrane Library, Medline, Scopus, ClinicalTrials.gov and reference lists of key articles, The search focused on recent studies published from January 2019 onward, with landmark studies considered when relevant. The search terms included intrahepatic cholangiocarcinoma, cholangiocarcinoma, tumour immune microenvironment, immune checkpoint inhibitor, immunotherapy, biomarker, FGFR2, IDH1, CAR‐T, CAR‐NK, dendritic cell vaccine and clinical trial. Studies were selected according to their relevance to ICC biology, clinical applicability, and mechanistic insights, with priority given to ICC‐specific clinical or translational studies, pivotal BTC trials involving ICC populations and recent mechanistic investigations. Evidence from other tumour types was considered when mechanistic insights relevant to ICC were provided. Clinical trial information was verified using ClinicalTrials.gov records and available publications, including the study population, intervention, primary endpoints and reported ICC‐related outcomes. As this was a narrative review based on the published literature, no original genomic, transcriptomic, or patient‐level datasets were reanalysed. Therefore, no additional bioinformatic quality control procedures or dataset‐specific analyses were performed.

## 2 CURRENT CLINICAL LANDSCAPE AND POTENTIAL RISKS OF IMMUNOTHERAPY FOR ICC

### 2.1 Checkpoint biology and immunotherapy rationale for ICC

Immunotherapy, represented by immune checkpoint inhibitors (ICIs), is a key breakthrough in the treatment of ICC. Therapeutic strategies based on the PD‐1/PD‐L1 pathway have been relatively advanced. Studies have shown that a higher density of PD‐1−EOMES−CD8+ effector T cells is associated with favourable survival in ICC.^22^ The PD‐1/PD‐L1 pathway maintains an immunosuppressive state in the TME by inhibiting T‐cell proliferation and reducing the secretion of IFN‐γ, TNF‐α, and IL‐2.^23^ PD‐L1‐positive TAMs may also promote T cell exhaustion, mainly by acting on activated effector T cells in later stages of the immune response. CTLA‐4 mainly affects the early stage of T cell activation, whereas PD‐1/PD‐L1 mainly regulates activated effector T cells during later immune responses. Therefore, a dual blockade may generate complementary antitumour effects.^20^, ^24^, ^25^

### 2.2 First‐Line chemoimmunotherapy evidence in advanced BTC and Its relevance to ICC

After gemcitabine/cisplatin was established as the standard chemotherapy backbone for advanced BTC, the phase III TOPAZ‐1 trial expanded the first‐line treatment landscape by incorporating durvalumab. In longer‐term TOPAZ‐1 update, durvalumab plus gemcitabine/cisplatin achieved longer overall survival than placebo plus gemcitabine/cisplatin, with updated median OS of 12.9 versus 11.3 months and an HR of 0.76.^26^, ^27^ These findings provide clinical support for first‐line immunochemotherapy in advanced BTC. For ICC, which was included within the broader BTC population, these findings are clinically relevant, while further analyses incorporating tumour location, molecular characteristics, and biomarkers may help refine the patient selection.

KEYNOTE‐966 further strengthens the clinical evidence that supports this strategy. In this phase III trial, pembrolizumab plus gemcitabine and cisplatin improved overall survival compared with chemotherapy alone in patients with advanced BTC (median OS, 12.7 vs. 10.9 months; HR, 0.83).^28^ Because nearly 60% of the enrolled patients had ICC and the intrahepatic subgroup showed a generally consistent survival trend, these findings support the clinical relevance of chemoimmunotherapy for ICC. Further studies integrating tumour location, molecular subtype and biomarker status may help refine the identification of patients most likely to benefit.

### 2.3 Potential risks of immunotherapy for intrahepatic cholangiocarcinoma

Although immunotherapy for advanced ICC has made considerable progress, durable benefits are not observed in all patients, and additional trials are needed to clarify its efficacy and applicable populations.^29^ The unique TME and immune escape mechanisms of ICC contribute to the heterogeneous responses to immunotherapy. CTLA‐4‐positive lymphocytes and FOXP3‐positive regulatory T cells are commonly observed in ICC and may weaken antitumour T‐cell immunity.^30^ Aetiology and baseline immune status may further influence treatment response, as CTLA‐4‐positive tumour‐infiltrating lymphocytes have been associated with poor prognosis in ICC patients with intrahepatic bile duct stones.^22^, ^31^ Clinical benefits are not universal and resistance should be considered using standardized terminology. The Society for Immunotherapy of Cancer (SITC) Immunotherapy Resistance Taskforce distinguishes between primary resistance, secondary (acquired) resistance and progression after treatment discontinuation for PD‐(L)1 blockade.^32^ In ICC, these resistance phenotypes may arise from overlapping mechanisms, including low immunogenicity or defective antigen presentation, T‐cell exclusion by desmoplastic stroma, myeloid‐cell‐ and Treg‐mediated suppression, loss of IFN‐γ pathway sensitivity, neoantigen editing, tumour‐cell signaling alterations, and compensatory checkpoint signaling. TIM‐3 upregulation has been implicated in resistance to PD‐1 blockade, but most mechanistic evidence remains preclinical or cross‐tumour and requires longitudinal ICC‐specific validation.^33^

**FIGURE 1:**
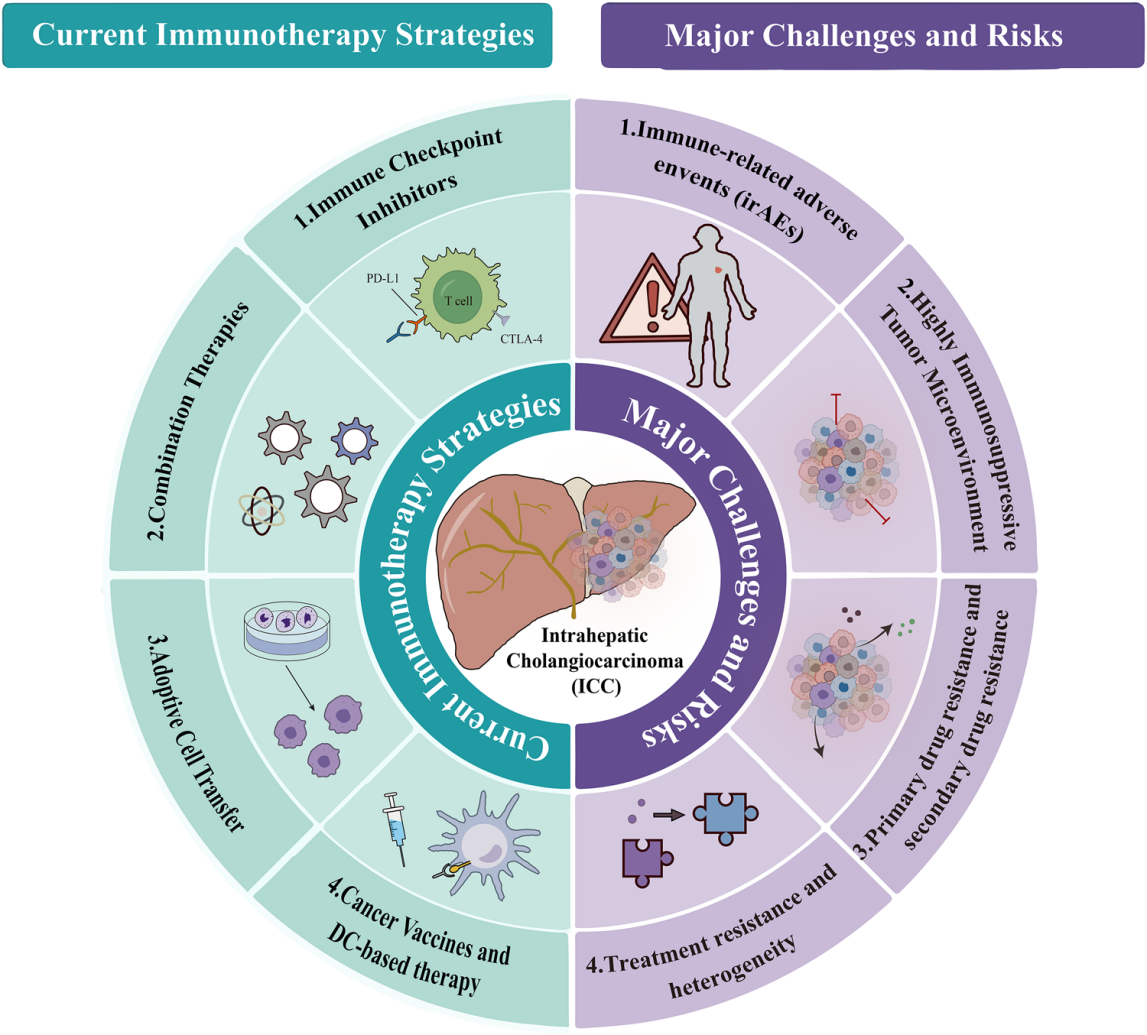
Current immunotherapy strategies and major challenges in ICC.

Immune‐related adverse events (irAEs) remain an important clinical consideration for ICI‐based therapy.^34^, ^35^ Broader ICI safety data indicate that toxicity patterns can vary across patient groups and that treatment‐associated electrolyte disturbances, including hyponatraemia, also warrant clinical surveillance.^36^, ^37^ Combination immunotherapy may enhance antitumour immunity but can also increase toxicity; therefore, treatment selection requires balancing expected benefits with toxicity risk and clinical feasibility (Figure 1).

In addition to TOPAZ‐1 and KEYNOTE‐966, several ICC‐focused or ICC‐relevant studies have explored immune‐based combinations. For a comparative analysis, Table 1 summarizes these trials according to treatment strategies, primary endpoints, available results and clinical interpretations.

**TABLE 1:** Clinical studies of immunotherapy strategies in ICC.

Experiment Name	Study design and population	Treatment strategy/ Main product	Primary endpoint and available result	Clinical interpretation	Reference
NCT04989218	Pilot/window‐of‐opportunity study in resectable or high‐risk ICC.	Gemcitabine/cisplatin plus durvalumab and tremelimumab.	The study primarily focused on safety and feasibility, with early clinical and pathological activity assessed as exploratory outcomes. Public information indicates trial termination, and limited safety data reported grade 3 vomiting and hypomagnesemia in one participant.	This perioperative ICC study provides useful feasibility and safety information, while its small and terminated status limits conclusions about efficacy.	^38^
NCT06058663	Phase I study in locally advanced unresectable ICC patients.	Yttrium‐90 (Y90) radioembolization combined with tremelimumab and durvalumab	The primary endpoint focused on dose‐limiting toxicity (DLT), with antitumour activity assessed as an exploratory outcome.	The study reflects the rationale of combining locoregional therapy with dual checkpoint blockade in ICC, with safety and dose‐finding as the current focus.	^39^
Experiment Name	Study design and population	Treatment strategy/ Main product	Primary endpoint and available result	Clinical interpretation	Reference
NCT03951597	Single‐arm phase II study in advanced unresectable ICC patients.	Oxaliplatin and gemcitabine chemotherapy, lenvatinib and anti‐PD‐1 antibody JS001/toripalimab	The primary endpoint focused on objective response rate (ORR). Published ICC data demonstrated preliminary activity and manageable safety, with interpretation limited by the absence of a randomized control group.	This is a clinically relevant ICC‐specific combination study and supports further evaluation of chemotherapy, targeted therapy and PD‐1 blockade in advanced ICC.	^40^
NCT06375642	Single‐arm phase II study in advanced ICC patients.	Adebrelimab plus surufatinib and hepatic‐artery infusion chemotherapy.	Planned endpoints include objective response rate (ORR) and disease control rate (DCR). Mature efficacy results had not been publicly reported.	The regimen combines immunotherapy, antiangiogenic or targeted therapy, and regional chemotherapy, representing an active combination strategy for advanced ICC.	^41^
NCT07147088	Single‐arm phase II study in advanced ICC after progression on GemCis plus PD‐1/PD‐L1 inhibitor.	Cryoablation with SHR‐1701 and famitinib.	The primary endpoint is objective response rate (ORR). Mature published efficacy results had not been publicly reported.	This study addresses subsequent‐line treatment after immunochemotherapy and integrates local ablation, PD‐L1/TGF‐β blockade and multitarget tyrosine kinase inhibition.	^42^
NCT05223816	Open‐label phase II study including ICC patients.	VG161 recombinant human IL12/15‐PDL1B oncolytic HSV‐1 injection (Vero cells)	Endpoints include safety, tolerability and early efficacy assessment, with mature ICC‐specific efficacy results had not been publicly reported.	The study illustrates oncolytic virus‐based immunotherapy in liver cancer or ICC‐relevant settings and may help inform future combination strategies.	^43^
NCT03201458	Interventional clinical trial in metastatic unresectable ICC patients.	Atezolizumab (PD‐L1 inhibitor), cobimetinib (kinase inhibitor)	The primary endpoint was PFS. In the published BTC phase II study, atezolizumab plus cobimetinib prolonged median PFS compared with atezolizumab alone, whereas objective responses remained limited.	This row supports the exploration of MEK inhibition plus PD‐L1 blockade in BTC. Its relevance to ICC is clinically informative but should be considered in the context of a mixed BTC population.	^44^

*Note*: Clinical trial information was obtained from ClinicalTrials.gov and available publications. This table summarizes clinical studies evaluating immunotherapy‐based strategies in ICC. Trials involving broader BTC populations are included when ICC‐specific analyses or clinical relevance are available. Early‐phase studies without mature efficacy data are interpreted according to their safety, feasibility, or exploratory objectives.

## 3 CHARACTERISTICS AND CROSSTALK OF THE ICC TUMOUR IMMUNE MICROENVIRONMENT

A defining feature of ICC is its highly desmoplastic stroma. ICC also arises within the liver, where baseline immunoregulatory features and resident immune populations provide a biological context that influences tumour–immune interactions. Rather than acting as a passive scaffold, CAF‐rich extracellular matrix (ECM), hypoxia, chronic inflammatory signaling, and myeloid‐cell recruitment interact dynamically to form a stromal barrier that restricts immune cell infiltration and promotes immune suppression. These stromal and immune components collectively form a regulatory network involving CAFs, MDSCs, TAMs, TANs, DCs, NK cells, and T cells, thereby contributing to immune escape and treatment resistance in ICC.

### 3.1 Myeloid immunosuppressive networks: MDSCs, TAMs and TANs

MDSCs exert immunosuppressive functions by inducing various factors and regulating multiple immune cells.^45^ They inhibit T‐cell proliferation and induce T‐cell apoptosis by regulating amino acid metabolism and secretion of ARG1 and iNOS.^46^, ^47^ Available ICC evidence suggests that MDSCs can enter ICC tumours through the CCL2/CCR2 pathway and suppress CD8+ T‐cell function. MDSCs secrete TGF‐β, reduce NKG2D expression, and impair NK cell activation.^48^ In addition, MDSCs can promote tumour growth through non‐immune mechanisms, such as enhancing cancer stem cell characteristics, promoting epithelial‐mesenchymal transition (EMT), promoting tumour angiogenesis and facilitating the formation of pre‐metastatic niches.^49^ In ICC, fibroblast activation protein‐positive CAFs may recruit MDSCs through the CCL2/CCR2 axis, and CAF‐derived IL‐6/IL‐33 can induce MDSCs to generate leukotriene B4, thereby promoting ICC stemness and tumour growth.^50^, ^51^ Taken together, these findings suggest that the immunosuppressive TIME of ICC is shaped by the coordinated interactions between myeloid cells, stromal components, lymphocytes and metabolic signals. Further ICC‐specific studies are required to clarify the influence of these regulatory networks on immune escape, treatment resistance and patient stratification.

TAMs have complex heterogeneity and multifunctionality and can be broadly classified into M1‐like macrophages, which inhibit cancer cells, and M2‐like macrophages, which promote tumour growth.^52^ TAMs in ICC mainly show M2‐like polarization and promote tumour proliferation, angiogenesis, metastasis and treatment resistance.^53^ M2‐like TAMs secrete IL‐10, TGF‐β, PGE2, VEGF and MMPs, which support immune suppression and stromal remodeling. In addition to their direct protumour effects, TAMs can reinforce CAF activation, MDSC accumulation, and Treg recruitment through cytokine and chemokine networks, thereby forming a myeloid‐stromal suppressive circuit that may reduce responses to ICIs and antiangiogenic therapy.

The role of TANs in the tumour immune microenvironment of ICC is dynamic and can be polarized into anti‐tumour N1 and pro‐tumour N2 types.^54^ Under immunostimulatory conditions, N1‐like TANs may support cytotoxic antitumor responses. In contrast, a TGF‐β‐rich, hypoxic, and chronically inflamed ICC microenvironment can promote N2‐like TAN polarization, which is associated with increased ARG1, VEGF, and immunosuppressive cytokines, leading to T‐cell suppression, angiogenesis, and tumour invasion.^55^ Thus, TAN polarization is mainly driven by TGF‐β‐dominated microenvironmental cues, whereas TANs and TAMs interact within the myeloid‐stromal suppressive axis. In ICC models, TAN‐derived OSM and TAM‐derived IL‐11 jointly activated STAT3 signaling in tumour cells, thereby enhancing ICC cell proliferation and invasion.^56^

### 3.2 Antigen presentation and innate immune surveillance: DCs and NK cells

DCs are specialized antigen‐presenting cells. In the ICC immune microenvironment, their infiltration is reduced and their distribution has specific characteristics. Evidence from human ICC samples indicates that mature CD83+ DCs are mainly located at the invasive margin, whereas immature CD1a+ DCs are more frequently detected within tumour tissues.^57^ A higher density of mature DCs is generally associated with stronger CD8+ T‐cell infiltration, suggesting that preserved antigen presentation may contribute to local antitumor immunity. In contrast, mechanistic studies, including evidence from other solid tumours, suggest that hypoxia, adenosine, IL‐10 and TGF‐β may promote tolerogenic DC phenotypes, thereby impairing antigen presentation, weakening T‐cell function, and supporting suppressive immune cell recruitment.^58^

NK cells are important components of the innate immune system and can non‐specifically recognize and attack cancer cells, thereby contributing to the control of tumour cell growth, migration and apoptosis. Recent ICC‐related studies have suggested that increasing the number or function of NK cells may prevent ICC progression. In vitro, arginine‐activated NK cells combined with an EGFR antibody can effectively kill ICC cell lines, and multiple infusions of ex vivo‐expanded human NK cells have shown antitumour activity in preclinical ICC settings.^59^ However, ICC cells may escape NK cell‐mediated killing through Fas/FasL‐related apoptosis induction and upregulation of anti‐apoptotic molecules such as c‐FLIP.^60^, ^61^ Because part of the NK cell rationale is derived from preclinical or cross‐tumour evidence, future ICC studies should evaluate the NK cell activation status, NKG2D expression, and suppressive cytokines in defined patient cohorts.

### 3.3 Adaptive lymphocyte axis: B cells, CD8+ T Cells, CD4+ T cells and tregs

Different subpopulations of tumour‐infiltrating B cells participate in the regulation of the tumour immune microenvironment (TIME) through distinct molecular mechanisms that not only exert humoral immune functions but also regulate the functions of various immune cells.^62^ Bulk transcriptomic and immune‐infiltration analyses have suggested that PNOC‐related B‐cell infiltration is associated with improved overall survival in ICC patients, and higher intratumoral GrB+ B‐cell infiltration has also been linked to a favourable prognosis in limited ICC cohorts.^63^, ^64^ However, these observations are mainly derived from retrospective sequencing or immunohistochemical analyses, and have not yet been validated as independent predictive biomarkers for immunotherapy. Therefore, B cell‐related signatures should be regarded as exploratory indicators of antitumour humoral immunity.

T cells in the TME of ICC exhibit various phenotypes, with CD8+, CD4+, and Treg cells playing a major role. CD8+ T cells are the major effector cells involved in antitumour immunity in ICC TIME. Among them, tissue‐resident CD103+CD8+ T cells have been reported to correlate with favourable prognosis in human ICC. particularly in tumours with a higher CD4+ T‐cell density and stronger CD8+ T‐cell infiltration. The higher the proportion of CD103+CD8+ T cells among all CD8+ T cells, the lower the possibility that ICC patients are at an advanced stage.^65^ Therefore, CD103+CD8+ T cells are considered promising exploratory prognostic indicators.

CD4+ T cells include multiple functional subpopulations that may exert dual effects on ICC. Th1 subpopulations enhance the recruitment of CD8+ T and NK cells by secreting IFN‐γ and TNF‐α, inhibiting angiogenesis, and promoting the M1 polarization of TAMs. In contrast, Th2 subpopulations promote tumour growth by secreting IL‐4, IL‐5, and IL‐13 and suppressing Th1‐related immune responses. Th17 subpopulations also play a dual role; however, in ICC, the protumour effect is dominant. Current immune cell atlas data suggest that the total CD4+ T‐cell density alone may be insufficient to predict overall survival, indicating that functional subsets and spatial distribution may be more informative than bulk CD4+ T‐cell abundance.^10^

Tregs are the major immunosuppressive lymphocytes enriched in the ICC TIME. Through FOXP3 overexpression, secretion of IL‐10 and TGF‐β, and high expression of inhibitory receptors such as CTLA‐4, Tregs suppress effector T‐cell activity and promote immune evasion.^8^ High Treg infiltration in ICC tissues is associated with chemotherapy resistance and poor patient prognosis. Overall, the balance between CD8+, CD4+, and Treg cells may shape the immune status of ICC, and should be considered when evaluating biomarkers and immunotherapy strategies.

### 3.4 Non‐cellular components and metabolic remodeling of the ICC time

**FIGURE 2:**
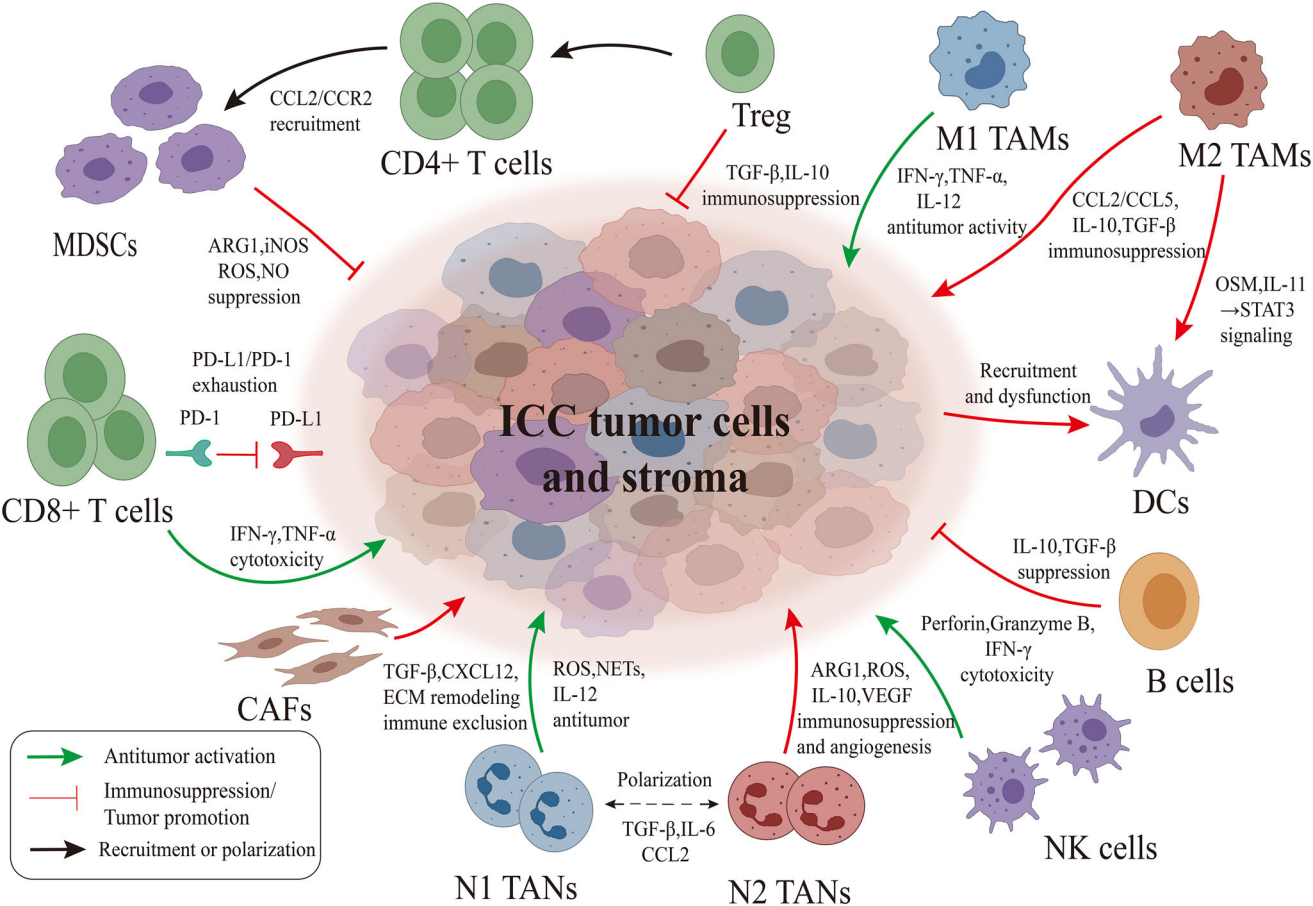
Immune microenvironment and cellular crosstalk in ICC.

Non‐cellular components, including the ECM, chemokines, extracellular vesicles (EVs), platelet‐derived growth factor (PDGF), and Notch signaling, contribute to the maintenance of the immunosuppressive ICC niche, and the desmoplastic ECM in ICC is not merely a structural scaffold but also an active regulator of tumour–immune interactions. Dense collagen deposition and matrix remodeling can modify tissue stiffness and interstitial properties, potentially restricting immune‐cell trafficking and promoting CAF activation. Fibrotic liver remodeling may reinforce this stromal barrier through macrophage‐myofibroblast crosstalk involving TGF‐β and PDGF signalling.^66^ Activated CAFs further remodel ECM components and release immunoregulatory factors, including TGF‐β, IL‐6, and chemokines, which may facilitate immune exclusion and recruitment or polarization of suppressive myeloid populations.^16^, ^17^, ^18^ In addition, chemokine networks, PDGF‐related signaling, extracellular vesicles, and Notch pathways provide additional mechanisms for communication between tumour cells and the surrounding microenvironment, thereby reinforcing immunosuppressive characteristics within the ICC niche.^19^ EV‐mediated transfer of regulatory molecules and Notch activation may further influence stromal remodelling, immune cell function, and tumour adaptation under therapeutic pressure. Metabolic remodeling represents another important layer of immune regulation in ICC. Hypoxia, lactate accumulation, arginine depletion, tryptophan metabolism, bile acid‐related signalling and IDH1‐mutant 2‐hydroxyglutarate may impair effector T‐cell function, antigen presentation, and NK‐cell cytotoxicity, thereby contributing to immune escape and resistance to immunotherapy.^67^ In AKT‐hyperactivated ICC, metabolic rewiring through the pPCK1‐pLDHA‐SPRINGlac axis has been linked to ferroptosis‐mediated chemo‐immunotherapy resistance.^68^ Collectively, alterations in non‐cellular components and metabolic states interact with cellular immune networks to shape the suppressive TIME of ICC and influence tumour progression and therapeutic responses (Figure 2).

## 4 MOLECULAR SUBTYPES, BIOMARKERS AND PRECISION‐IMMUNOTHERAPY IMPLICATIONS

ICC is a highly heterogeneous type of malignant tumour, and this heterogeneity manifests in various aspects such as anatomical location, histological type, molecular alterations, immune infiltration and treatment sensitivity. FGFR2 fusion or rearrangement and IDH1 mutations are representative alterations in ICC, and changes in BAP1, ARID1A, KRAS and TP53 may also affect the biological behaviour of the tumour.^69^ Recent immune profiling further showed that small duct‐ and large duct‐type ICC have distinct immune signatures, suggesting that histological and molecular subtypes may also be linked to different immune states.^70^ Therefore, when formulating individualized treatment strategies for ICC, anatomical classification, histological subtype, genomic alterations, and immune status should be considered comprehensively.

FGFR2‐ and IDH1‐altered ICC has clear clinical relevance in molecular‐targeted therapies. For IDH1‐mutant cholangiocarcinoma, the phase III ClarIDHy trial showed that ivosidenib improved progression‐free survival compared to placebo.^71^ In FGFR2 fusion‐ or rearrangement‐positive cholangiocarcinoma, pemigatinib showed antitumour activity in the FIGHT‐202 study, with an objective response rate of approximately 37% and median progression‐free survival of 7.0 months in the final analysis.^72^ Futibatinib also showed clinically meaningful activity in FGFR2 fusion‐ or rearrangement‐positive ICC in the FOENIX‐CCA2 study, with an objective response rate of 42% and median progression‐free survival of 9.0 months.^73^ These targeted‐agent data support molecular stratification and treatment selection in ICC; however, their immunotherapy implications require separate evaluation through immune profiling and prospective combination studies.

The relationship between molecular alterations and TIME is also important for precision immunotherapy; however, current evidence remains incomplete. Genomic and transcriptomic analyses suggest that different BTC and ICC subtypes may exhibit distinct patterns of DNA alterations, inflammatory transcriptional programs, and immune‐related features.^74^ IDH1‐mutant tumours may influence immune regulation through 2‐hydroxyglutarate‐related metabolic remodelling, which could affect immune cell function and the local immune environment.^67^ Similarly, FGFR2‐altered ICC may display differences in immune cell infiltration and immune checkpoint expression, suggesting a potential link between molecular subtype and immune contexture.^75^ BAP1/ARID1A‐and KRAS/TP53‐related molecular states may represent additional biologically distinct contexts; however, their ability to predict ICI response has not been prospectively validated. Therefore, molecular alterations should be considered as a hypothesis‐generating framework that requires integration with spatial and functional immune profiling rather than as independent predictors of immunotherapy efficacy.

The identification of predictive biomarkers is essential for improving ICC immunotherapy. However, clinically actionable biomarkers should be distinguished from exploratory candidates. MSI‐H/dMMR and, in some regulatory settings, high tumour mutational burden (TMB) can support tissue‐agnostic ICI treatment selection; however, these abnormalities are uncommon in ICC and should not be considered as ICC‐specific biomarkers. In contrast, PD‐L1 expression has shown limited discriminatory value in BTC, with clinical responses reported in both PD‐L1‐positive and PD‐L1‐negative groups.^76^ FGFR2/IDH1 status, BAP1/ARID1A alterations, immune‐cell infiltration patterns, tertiary lymphoid structures, circulating inflammatory indicators, and metabolic characteristics should currently be regarded as exploratory candidates for predicting ICI benefit.^69^, ^77^, ^78^ Systemic metabolic states may also confound circulating metabolic biomarkers, as obesity‐associated glucose dysregulation is accompanied by substantial shifts in serum lipidomic profiles.^79^ Importantly, FGFR2 and IDH1 alterations are clinically validated biomarkers for targeted therapy, rather than established biomarkers for immunotherapy selection. Therefore, multimodal strategies integrating genomic information, spatial immune context, and dynamic treatment response features may provide a more comprehensive assessment of immunotherapy sensitivity than any single biomarker alone.

Based on these features, precision immunotherapy for ICC may evolve beyond simple checkpoint blockade to more stratified and mechanism‐informed treatment approaches. Currently, immune‐inflammatory, immune‐excluded, and myeloid‐cell‐dominant categories should be considered as conceptual frameworks for describing ICC immune phenotypes rather than established therapeutic classifications. Immune‐inflammatory tumours may represent more favourable contexts for ICI‐based combinations, whereas immune‐excluded tumours may require strategies targeting stromal barriers, CAF/TGF‐β/ECM‐related signaling, or angiogenic pathways, which may benefit from approaches involving TAM or MDSC modulation. However, these strategies remain under investigation. Targeted therapies have demonstrated clinical efficacy for FGFR2‐ or IDH1‐altered tumours; however, whether pathway inhibition can consistently reshape immune‐cold or immune‐excluded phenotypes to enhance ICI sensitivity remains unclear. Therefore, targeted therapy/ICI combinations should currently be regarded as biomarker‐guided clinical hypotheses requiring prospective evaluation rather than routine treatment recommendation.^6^, ^7^, ^71^, ^72^, ^73^ Similarly, the immunotherapeutic implications of BAP1/ARID1A and other exploratory genomic states require further validation. A practical research framework involves integrating molecular characteristics with spatial immune profiling, identifying dominant resistance mechanisms, and developing biologically matched combination strategies.

## 5 EMERGING IMMUNOTHERAPY STRATEGIES FOR ICC

### 5.1 Novel immune checkpoint inhibitors for ICC

In addition to PD‐1/PD‐L1 and CTLA‐4 inhibitors discussed above, several emerging immune checkpoint targets, including LAG‐3, TIM‐3, B7‐H3, and TIGIT, have attracted increasing attention. LAG‐3 inhibition alone has shown limited activity in many clinical settings, whereas combined PD‐1/LAG‐3 blockade may enhance antitumour immunity and has demonstrated efficacy in selected tumour types.^80^ The LAG‐3 antibody LBL‐007 combined with tislelizumab and chemotherapy has shown activity in advanced nasopharyngeal carcinoma,^81^ providing a biological rationale for further investigation rather than direct evidence for ICC treatment. Similarly, TIM‐3‐directed approaches combined with PD‐1/PD‐L1 blockade and B7‐H3‐targeted strategies have entered early phase clinical evaluation in selected tumours.^82^, ^83^ In ICC, these emerging checkpoints should be considered exploratory strategies for overcoming immune resistance. Future studies are needed to determine appropriate patient selection based on immune infiltration patterns, prior ICI exposure, molecular characteristics and target expression, rather than assuming efficacy based on findings from other tumour types.

### 5.2 ADCs and bispecific antibodies as antigen‐directed strategies

Antibody–drug conjugates (ADCs) are promising antigen‐directed therapeutic agents for tumour treatment. Two ICAM1‐targeted ADCs, ICAM1‐DXd and ICAM1‐MMAE, were evaluated using cholangiocarcinoma models or samples. The results showed inhibition of tumour growth, and analysis of 78 patients suggested that a subset of ICC tumours express ICAM1 and may be candidates for ICAM1‐directed strategies.^84^ Bispecific antibodies (BsAbs) targeting HER2 × CD3, including M802 and GBR1302, have been evaluated in preclinical models and HER2‐positive solid tumors.^85^, ^86^ These observations may be relevant to molecularly selected ICC with high target expression, and the therapeutic relevance of target expression for patient selection requires further investigation. Antigen heterogeneity, heterogeneous target density, target loss, on‐target/off‐tumour toxicity, and limited drug or immune cell penetration through the desmoplastic stroma may reduce efficacy. Thus, HER2‐directed activity from other tumour types or preclinical ICAM1 findings should be regarded as preliminary evidence supporting further investigation, rather than established ICC treatment efficacy.

### 5.3 CAR‐T‐cell therapy

Chimeric antigen receptor therapy has achieved great success in haematological malignancies and has promoted research on solid tumours.^87^ Mao et al. developed CAR‐T cells targeting Tn‐MUC1‐positive ICC and demonstrated in preclinical experiments that these cells specifically eliminated Tn‐MUC1‐positive ICC cells without affecting Tn‐MUC1‐negative ICC cells in vitro and in animal models.^88^ Nebbia et al. studied CAR‐T cell therapy targeting B7‐H3 and showed that B7‐H3 CAR‐T cells inhibited ICC growth in mice and prolonged their survival.^89^ Qiao et al. designed multi‐target CAR‐T cells targeting multiple immune mechanisms including PTG‐T16R‐scFv‐CAR‐T cells. They selected three highly expressed immune checkpoints (PD‐1, TIGIT, and TIM‐3) and three key immunosuppressive cytokine receptors (TGFβR, IL‐10R, and IL‐6R) from CCA tissue, and both in vitro and in vivo experiments demonstrated that this therapy inhibited CCA tumour growth.^90^ CAR‐T cell therapy can target single or multiple antigens and has shown promise mainly in preclinical ICC models. However, the translation of CAR‐T strategies into solid tumours remains challenging owing to limited T‐cell infiltration, persistence, and survival within the TME, as well as the immunosuppressive and heterogeneous nature of solid tumour environments.

### 5.4 CAR‐M and CAR‐NK therapies

CAR‐M therapy involves modifying macrophages to convert them from an M2‐like phenotype to an M1‐like phenotype, enabling them to phagocytose tumour cells, present antigens, and activate T‐cell immunity.^91^ A HER2‐targeted CAR‐M product, CT‐0508, is being evaluated in HER2‐overexpressing solid tumours, which provides a rationale for exploring CAR‐M strategies in molecularly selected ICC populations with appropriate HER2 expression, although their relevance to ICC requires further investigation.^92^ CAR‐NK therapy combines the innate cytotoxicity of NK cells with CAR‐guided antigen recognition. Current clinical trials are exploring CAR‐NK cells that target NKG2D, Claudin 6, carcinoembryonic antigen, 5T4, ROBO1 and MUC1. Recent advances support the potential of CAR‐NK as an off‐the‐shelf platform; however, its application in ICC remains an emerging research direction.^93^, ^94^ CAR‐M cells may have advantages in tumour infiltration and microenvironmental remodeling, whereas CAR‐NK cells may offer favourable manufacturing and safety characteristics.^95^, ^96^ Nevertheless, target heterogeneity, cell persistence, large‐scale manufacturing and the limited availability of ICC‐related clinical evidence remain important challenges for both approaches.

### 5.5 Cancer vaccines and dendritic cell therapy for intrahepatic cholangiocarcinoma

**FIGURE 3:**
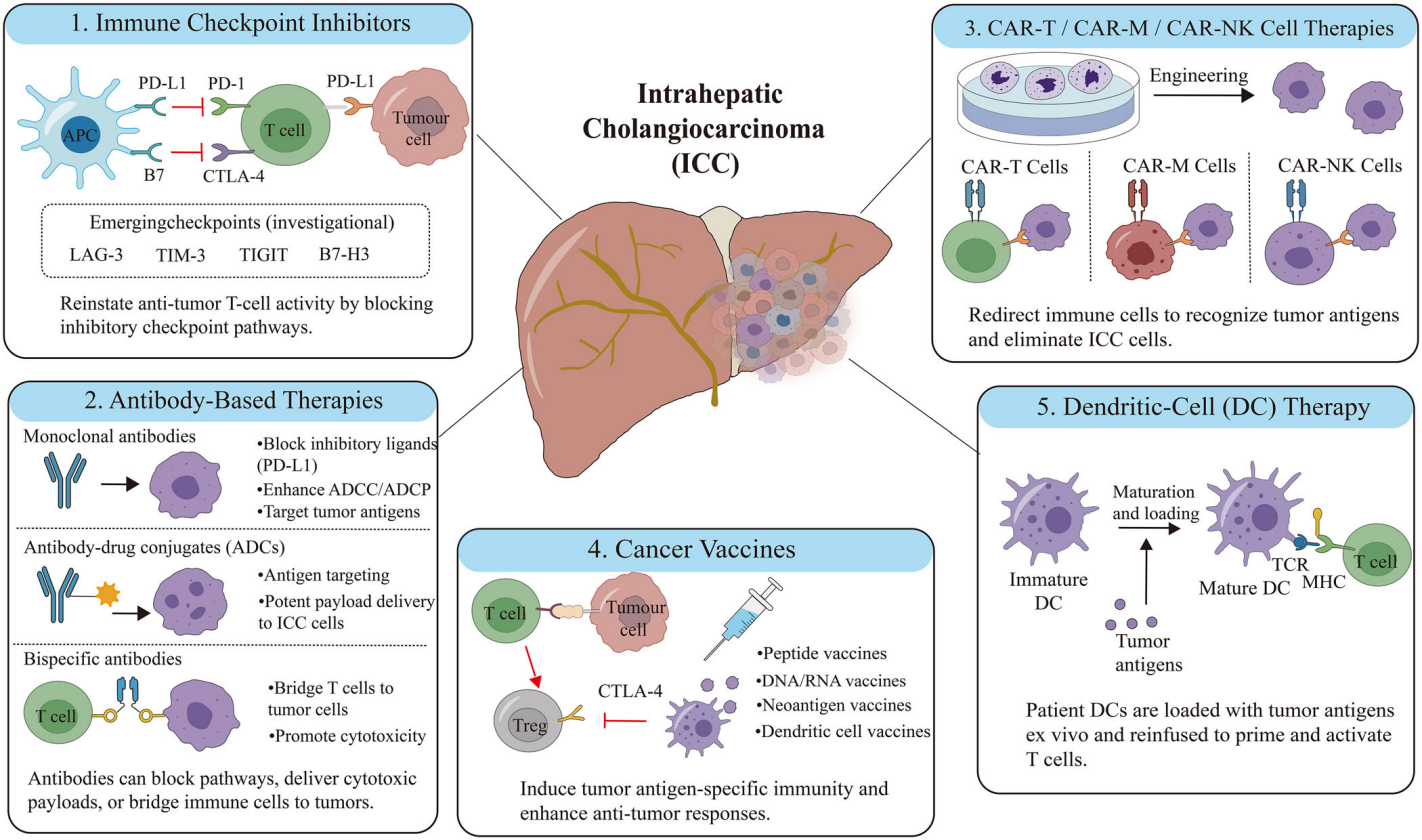
Emerging immunotherapeutic strategies for ICC.

Cancer vaccines aim to induce persistent and specific antitumor immune responses in patients, and can be divided into preventive and therapeutic vaccines. Therapeutic vaccines, such as dendritic cell (DC) vaccines, are mainly based on tumour antigens and may be used as adjuvant therapies after radiotherapy, chemotherapy, or surgical resection.^97^ DC vaccines use the antigen‐presenting ability of DCs to activate T‐cell functions. A clinical trial used DC vaccines loaded with autologous tumour lysates in combination with ex vivo activated T‐cell transfer (ATVAC) as adjuvant therapy in patients with ICC after surgery. The results showed that surgery plus ATVAC improved recurrence‐free survival (RFS) and overall survival (OS).^98^ However, the interpretation of these findings requires consideration of the study design and treatment‐era factors, and further validation is required before defining their clinical benefits. Personalized or mRNA vaccines based on ICC neoantigens are conceptually attractive; however, neoantigen quality, HLA restriction, impaired antigen presentation, individualized manufacturing time, and suppressive TIME remain important barriers. Future studies should clarify patient selection, vaccine design, and strategies to overcome immune suppression in ICC‐specific settings (Figure 3).

To provide an overview of emerging immunotherapeutic approaches in ICC, Table 2 summarizes ongoing, early‐phase, or ICC‐relevant clinical trials evaluating novel immunotherapeutic strategies.

**TABLE 2:** Emerging therapeutic approaches targeting immune regulation and tumour biology in ICC.

Experiment Name	Study design and population	Treatment strategy/ Main product	Primary endpoint and available result	Clinical interpretation	Reference
NCT06583993	Single‐arm open‐label pilot /early phase study in advanced ICC patients.	Intravenous HNF4α srRNA.	The primary endpoints focus on safety and tolerability, with preliminary activity assessed as an exploratory outcome. mature efficacy results had not been publicly reported.	This study represents an early translational strategy in ICC and is best understood as safety‐oriented exploration before efficacy can be established.	^99^
NCT03849469	Phase I dose‐escalation and expansion study in selected advanced solid tumors.	XmAb22841 as a monotherapy (targeting LAG3 and CTLA4).	The primary endpoint focuses on safety and tolerability, including treatment‐related adverse events and dose‐limiting toxicities. This trial enrolled multiple solid tumor subtypes including ICC, yet no mature ICC‐specific efficacy results have been publicly reported as the study was early terminated.	This study supports the broader development of novel checkpoint combinations. For ICC, it provides conceptual relevance rather than disease‐specific clinical efficacy.	^100^
NCT03633773	Phase I/II study in MUC‐1‐positive inoperable ICC patients.	MUC‐1 CAR‐T cells.	The study evaluates safety and antitumour activity, including adverse events and response‐related outcomes. Disease control rate (DCR) was reported as an efficacy outcome. This trial with a small planned enrollment of roughly 9 patients; No mature published ICC‐specific efficacy results are available.	This is an ICC‐relevant adoptive cell therapy study. The most balanced description is early‐phase evaluation of feasibility, safety and preliminary activity.	^101^
NCT03942328	Early‐phase study in unresectable primary liver cancer, including ICC‐relevant settings.	Intratumoral autologous dendritic cells with Prevnar and immune checkpoint inhibitors after radiotherapy.	The primary endpoint focuses on significant toxicity within two treatment cycles. The study remains open for recruitment, and mature clinical outcome data are not yet available.	The study connects radiotherapy, antigen presentation and checkpoint blockade, and is relevant to vaccine/DC‐based strategies aimed at strengthening antitumor immunity	^102^
NCT06406816	Single‐arm, open‐label exploratory study in ICC patients after radical resection with high recurrence risk.	Neoantigen vaccine plus capecitabine.	The study evaluates efficacy and safety in the adjuvant setting, with recurrence‐related outcomes assessed as exploratory observations. Mature recurrence and survival data are not yet available.	This study is closely related to precision immunotherapy because it links postoperative recurrence prevention with individualized neoantigen vaccination.	^103^
NCT04708067	Phase I window‐of‐opportunity study in advanced ICC patients.	Focal radiotherapy plus bintrafusp alfa.	The primary endpoint focuses on adverse events within 60 days. The study remains active but not recruiting, and mature clinical outcome data are not yet available.	The study is mechanistically relevant to ICC because it combines radiotherapy with simultaneous PD‐L1 and TGF‐β pathway inhibition, two processes closely linked to immune suppression and stromal regulation.	^104^

*Note*: This table summarizes emerging therapeutic approaches with potential relevance to immune regulation and precision treatment of ICC. Strategies include immune‐based therapies, cellular immunotherapies, and molecular approaches that may reshape tumor biology or enhance therapeutic responses. The current developmental stage and clinical applicability are interpreted according to available preclinical and clinical evidence.

## 6 DISCUSSION AND CONCLUSION

This review summarizes the complex composition of the ICC immune microenvironment and its central role in immune escape, with an emphasis on immune cell crosstalk, stromal barriers, metabolic remodeling, and non‐cellular components. It also reviews the current immunotherapy evidence, molecular subtype considerations, biomarker limitations, and emerging strategies. By integrating clinical evidence, TIME regulation, and molecular subtype information, this review aimed to support the development of individualized and mechanism‐guided treatment strategies for ICC.

Although immunotherapy has expanded the treatment landscape of ICC, several key challenges remain. The dynamic network of the ICC immune microenvironment is shaped by the synergistic inhibitory effects of TAMs, MDSCs, Tregs, and other cellular components as well as stromal, metabolic, and extracellular‐matrix‐related non‐cellular components. Metabolic remodelling further participates in immune escape and may influence treatment response.^67^ These factors contribute to heterogeneous treatment responses, primary or acquired resistance, and limited predictive value of single biomarkers. At the clinical level, much pivotal data still comes from mixed BTC cohorts, whereas ICC has distinct anatomical, molecular and immune characteristics.^105^

These observations highlight the need to integrate molecular subtypes, spatial immune contexts and dynamic biomarker monitoring to better define patient selection and treatment strategies for ICC immunotherapies.

The molecular heterogeneity of ICC should be considered in future immunotherapeutic designs. FGFR2 fusion or rearrangement, IDH1 mutations, BAP1/ARID1A alterations, and KRAS/TP53‐related subtypes reflect different biological backgrounds. Small and large duct‐type ICC may also have different molecular and immune characteristics.^69^, ^78^ Future research should integrate molecular typing with immune phenotypes and dominant resistance mechanisms to guide rational combination strategies. Recent studies on TME‐oriented molecular diagnosis, metabolic immune regulation and cellular immunotherapy may provide useful directions for mechanism‐guided ICC immunotherapy.^77^, ^106^

In addition to existing immune checkpoint therapies, ADCs, bispecific antibodies, CAR‐T/M/NK therapies, cancer vaccines, and dendritic cell‐based strategies provide new directions for ICC treatment, and their clinical roles in ICC continue to be explored. Among these emerging approaches, cell therapies and vaccine‐based strategies are closely related to future immunotherapies. Recent progress in CAR‐NK therapy for solid tumours supports its potential as an off‐the‐shelf cellular immunotherapy platform and may provide useful information for future ICC‐related explorations.^94^ However, these approaches should be evaluated through mechanism‐guided and biomarker‐informed studies, as most remain at the early clinical or experimental stages. Liver organoid models incorporating immune and stromal components may offer more physiologically relevant platforms for preclinical evaluation of biomarker‐guided immunotherapy.^107^

**FIGURE 4:**
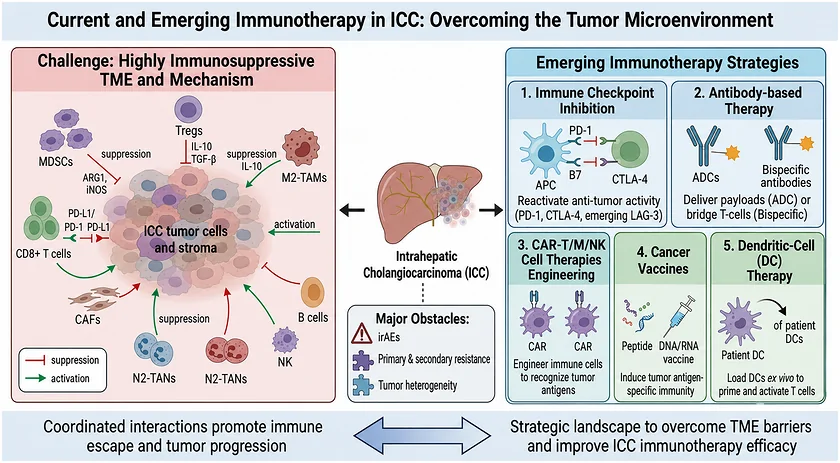
Current and emerging immunotherapy in ICC: overcoming the tumour microenvironment.

In the future, precision immunotherapy based on molecular typing and individualized neoantigen vaccines will remain an important direction for ICC research. FGFR2‐ and IDH1‐altered ICC have already established targeted treatment evidence, which may provide a rational basis for future molecularly selected combination trials.^71^, ^72^, ^73^ Therefore, combining molecular subtypes, immune phenotypes and effective biomarkers may gradually advance ICC immunotherapy from a broad combined treatment to a more personalized and mechanism‐based model. A deeper understanding of the ICC immune microenvironment is fundamental to the development of effective therapies. Further exploration of dynamic TIME regulation, validation of effective biomarkers, and development of rational combination strategies may help improve the poor prognosis of ICC patients and prolong their survival.^67^, ^77^ Simultaneously, therapeutic efficacy should be balanced with immune‐related adverse events and long‐term safety so that precision immunotherapy can be translated into clinical practice^21^ (Figure 4).

## AUTHOR CONTRIBUTIONS

**Mengyao Chen**: Writing the original draft, software, formal analysis, Investigation, visualization, data curation. **Shuofan Jin**: Methodology, software, validation, data curation, visualization, writing—original draft, writing—review and editing. **Yuxuan Zhou**: Methodology, software, validation, data curation, visualization, writing–original draft, and writing–review and editing. **Jinkai Tong**: Methodology, software, validation, data curation, visualization, writing–original draft, and writing–review and editing. **Yufan Zhao**: Methodology, software, validation, data curation, visualization, writing–original draft, and writing–review and editing. **Quanjun Yang**: Supervision, writing—original draft, writing–review and editing, conceptualization, project administration, and funding acquisition.

## CONFLICT OF INTEREST STATEMENT

The authors declare no conflicts of interest.

## CONSENT TO PARTICIPATE

This article is a review of previously published literature and does not include any new studies involving human participants or animals performed by any of the authors.

## ETHICS STATEMENT

The authors have nothing to report.

## Data Availability

No data were used for the research described in the article.
